# Fully closed-loop systems: can people with type 1 diabetes just do it? Insights from open-source systems

**DOI:** 10.1007/s00125-025-06644-8

**Published:** 2026-01-19

**Authors:** Rayhan Lal, Katarina Braune, Dana M. Lewis, Lenka Petruzelkova, Martin de Bock, Sufyan Hussain

**Affiliations:** 1 Departments of Medicine & Pediatrics, Division of Endocrinology, Stanford University, Stanford, CA USA; 2 Stanford Diabetes Research Center, Stanford University, Stanford, CA USA; 3 Hasso Plattner Institute for Digital Engineering, University of Potsdam, Potsdam, Germany; 4 Charité – Universitätsmedizin Berlin, Institute of Medical Informatics, Berlin, Germany; 5 OpenAPS, Seattle, WA USA; 6 Department of Pediatrics, Motol University Hospital and 2nd Faculty of Medicine, Charles University in Prague, Prague, Czech Republic; 7 Department of Paediatrics, University of Otago, Christchurch, New Zealand; 8 Department of Diabetes, School of Cardiovascular, Metabolic Medicine and Sciences, King’s College London, London, UK; 9 Department of Diabetes and Endocrinology, Guy’s & St Thomas’ NHS Foundation Trust, London, UK; 10 Institute of Diabetes, Endocrinology and Obesity, King’s Health Partners, London, UK

**Keywords:** AID, Automated insulin delivery, Diabetes technology, DIY, Fully closed-loop, Hybrid closed-loop, Open-source, Patient-led innovation, Review, Unannounced meals

## Abstract

Automated insulin delivery (AID) systems have significantly advanced diabetes management, progressively reducing user interactions required for optimal glucose management. This review evaluates the current landscape and future potential of AID systems without meal announcement, particularly focusing on real-world insights from open-source AID (OS-AID) technologies. Although commercial AID systems operating in hybrid closed-loop (HCL) mode have improved glycaemic outcomes, they remain dependent on manual meal announcement and user-driven actions, limiting their real-world utility. Current versions of OS-AID systems, developed by the diabetes community, can allow operation without meal announcements, presenting an opportunity to move closer to truly automated diabetes management. Recent clinical trials suggest that OS-AID systems can effectively manage glucose levels without meal announcements, achieving glucose levels comparable with those obtained by AID systems in HCL mode, with the potential of reduced management burden for users. However, practical challenges persist, including the need for expert configuration and handling of rapid changes in insulin sensitivity, such as during exercise or rapid glucose fluctuations following predicted low-glucose. This review synthesises insights from user and healthcare professional experiences, and emerging clinical evidence. It highlights the fact that successful implementation of AID without meal announcement requires advanced algorithmic responsiveness, user personalisation and ongoing clinician engagement. Looking forward, integrating adjunctive therapies, artificial intelligence and enhanced physiological modelling will likely enhance system performance to drive the next generation of diabetes care towards wider adoption and true ‘set-and-forget’ functionality.

**Graphical Abstract:**

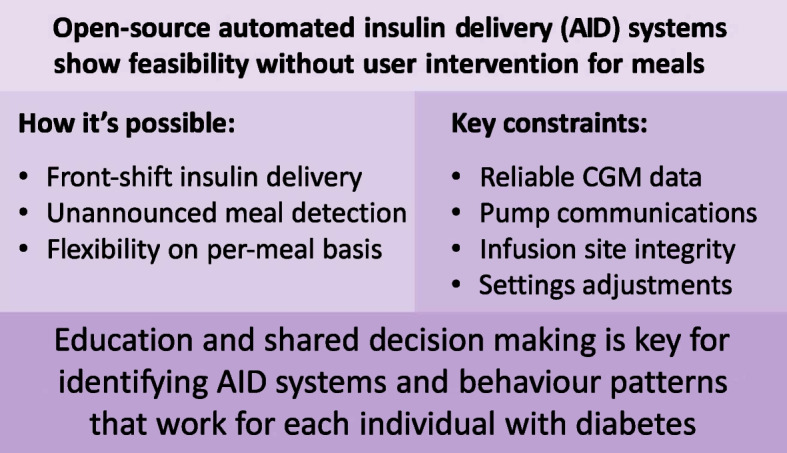

**Supplementary Information:**

The online version contains peer-reviewed but unedited supplementary material including a slideset of the figures for download, available at 10.1007/s00125-025-06644-8.

## Introduction

The advent of automated insulin delivery (AID) has significantly transformed diabetes management. Over the past decades, technologies have progressively evolved from continuous subcutaneous insulin infusion (CSII) to sensor-augmented pump therapy (SAPT) and predictive low-glucose suspend (PLGS) systems. These developments have reduced the burden of hypoglycaemia by suspending insulin delivery in response to predicted lows, paving the way for systems with increased degrees of automation [1].

The next step in automation was delivering additional insulin for glucose above (or predicted to be above) target. These systems, however, still required user-provided inputs, particularly for meals and physical activity. This led to the development of ‘hybrid closed-loop’ (HCL) systems, this term now being widely used to describe systems that automate basal rate adjustments and occasional corrections but depend on manual input for key events. While the form of meal and exercise announcement varies by system, all commercial AID options currently require quantitative or qualitative carbohydrate entry for the most optimal glucose outcomes.

The term ‘fully closed-loop’ (FCL) remains contested. Some use it to describe systems operating without meal announcements or manual boluses while others reserve it for systems requiring no user input at all. Still others argue that FCL should only be used to describe systems with no human involvement in operation or oversight. AID systems without meal announcement may still require manual input (e.g. exercise announcement) and therefore could even be labelled HCL. Given the imprecision of the term FCL, we will explicitly describe AID systems without meal announcement as systems that remove the need for user interaction for meals. It is important to define this from the user perspective, where this progression may appear as highlighted in Table 1. It is crucial to understand that the explicit implementation of these technologies is not necessarily tied to our current AID approaches involving continuous glucose monitoring (CGM), insulin pump and algorithm. For example, a true FCL system could involve depots of glucose-sensitive insulin.

**Table 1:** User-centred progression towards FCL systems for s.c. AID

Progression towards FCL	User perspective
SAPT	I look at my glucose continuously and manage all aspects of insulin delivery (hypoglycaemia, hyperglycaemia, meals, exercise/changes in ISF, equipment failure)
LGS/PLGS	The system alleviates my burden of hypoglycaemia by withholding insulin for predicted hypoglycaemia but I must still manage hyperglycaemia, meals, exercise/changes in ISF, and equipment failure
HCL	The system alleviates my burden of hypoglycaemia by withholding insulin for predicted hypoglycaemia It also reduces my burden of hyperglycaemia management by increasing insulin delivery but I must still announce meals, exercise, changes in insulin sensitivity and equipment failure
AID without meal announcement	The system alleviates some of my burden of hypoglycaemia by withholding insulin for predicted hypoglycaemia and it can increase insulin delivery sufficiently for meals and other predicted hyperglycaemia but I must still manage exercise, changes in insulin sensitivity and equipment failure
AID without any announcement	The system maintains my glucose in a wide variety of situations, including exercise, stress and illness and alleviates my burden of hypoglycaemia and hyperglycaemia, but I must still manage equipment failure
FCL	I can ‘set-and-forget’ every few days There is no need for continued interaction with my diabetes technology to maintain my health, other than occasional equipment failure

This table illustrates how different stages of subcutaneous AID are perceived by the user, highlighting the gradual reduction in user input required for managing their diabetes. It reflects the ambiguity surrounding the term ‘FCL’ and clarifies distinctions based on user experience rather than solely technical features. A broad range of implementations are possible for each step. LGS, low-glucose suspend

A driving force towards full automation has been the #WeAreNotWaiting community, which created open-source AID (OS-AID) systems having algorithms and other code that are open-source and available for anyone, including users, to see and understand [2]. OS-AID systems using the OpenAPS algorithm currently provide the only real-world option for insulin delivery without meal announcements [3, 4]. The progression from PLGS to HCL and AID without meal announcement reflects not only a technological evolution but also a philosophical shift towards algorithm-driven decision-making over behavioural expectations placed on users.

This review focuses specifically on AID systems that operate without meal announcements, an area where evidence is now emerging. We examine the evolution of such systems, emphasising real-world insights from the OS-AID community. These experiences can help guide future development and inform the design of next-generation AID systems.

## Experience of commercial AID systems

Since the introduction of the Medtronic 670G system, several commercial AID systems have become available [1]. Clinical outcomes from these systems consistently demonstrate improvements in glucose levels, reductions in hypoglycaemia and decreased mental burden compared with traditional multiple daily insulin injections (MDI) or SAPT without automation [5, 6]. However, achieving time in range (TIR) consensus goals with these AID systems relies on meal announcements [7].

Despite delivering substantial improvements in glycaemic outcomes, current commercial AID systems often fall short of achieving the widely accepted international glycaemic targets for many users, and optimal time-in-target often requires a lot of effort and user engagement [8–11]. This discrepancy is evident in real-world data reflecting the practical challenges faced by users, particularly among younger populations and those experiencing a high bolus burden [7, 12, 13]. In these groups, maintaining consistent meal-time insulin bolus entries and accurate carbohydrate estimates is frequently problematic, highlighting constraints in glycaemic attainment.

These limitations can become more pronounced within public healthcare systems, where social determinants of health and demographic diversity may further exacerbate barriers to optimal diabetes management and achievement of international glycaemic targets [14–17]. Consequently, while commercial AID systems provide significant improvements in glucose levels and can achieve this at a population level through widescale implementation efforts, real-world usage underscores significant challenges in effectively achieving optimal glycaemic outcomes for a significant proportion of individuals, with more effective meal management required to improve their limitations [18].

Pilot trials of commercial AID systems, involving controlled meals without prior bolus or meal entry, reinforce these challenges, demonstrating difficulties in maintaining optimal glycaemic management under these circumstances [19–21]. Although many commercial algorithms remain trade secrets, clinical observations indicate that commercial algorithms have limitations in their responsiveness to unexpected glucose fluctuations. The prevalent ‘one-size-fits-all’ design philosophy in current commercial AID systems simplifies usage but restricts the capacity for personalised algorithm adjustment. Additionally, the requirement for manual interventions following limited or manual modes, or forced calibrations in response to persistently elevated glucose levels, in some AID systems further highlights limitations when regular meal-time insulin and carbohydrate entries are not consistently executed. Given these recognised limitations, ongoing development and clinical evaluation of next-generation commercial systems now increasingly aim to achieve AID without meal announcement, promising enhanced adaptability and broader applicability in real-world diabetes care [21].

## Experience of OS-AID systems

OS-AID systems were first developed by people living with diabetes and their loved ones, years before commercial systems became available anywhere in the world [2, 22]. They were first developed using off-the-shelf consumer hardware devices (e.g. OpenAPS ‘rigs’), with the algorithms later being ported to smartphones running Android (AndroidAPS, now known as AAPS, which uses the OpenAPS algorithm) and iOS (Loop, which uses its own specific algorithm, and Trio/iAPS, which uses the OpenAPS algorithm) [23–27]. OS-AID systems were first developed as HCL systems, similar to first- and second-generation commercial systems, relying on carbohydrate announcements and manual meal boluses from users. However, users soon began requesting additional features to help combat postprandial glycaemic excursions. In response, the ‘oref1’ OpenAPS algorithm was extended to include supermicroboluses (SMBs) and unannounced meals (UAMs). SMBs enable more rapid delivery of insulin by using small boluses combined with a reduction of basal insulin in order to front-shift insulin activity. UAMs have enabled the algorithm to more quickly adjust insulin delivery to unexpected glucose excursions even in the absence of carbohydrate announcements. Both are part of the OpenAPS algorithm family*.* Further developments include dynamic insulin sensitivity factors (DynISF), which automatically adjust insulin sensitivity based on total daily dose (TDD) and real-time CGM trends, with some model-based algorithms (e.g. iAPS) additionally incorporating predicted CGM trajectories.

**Fig. 1:**
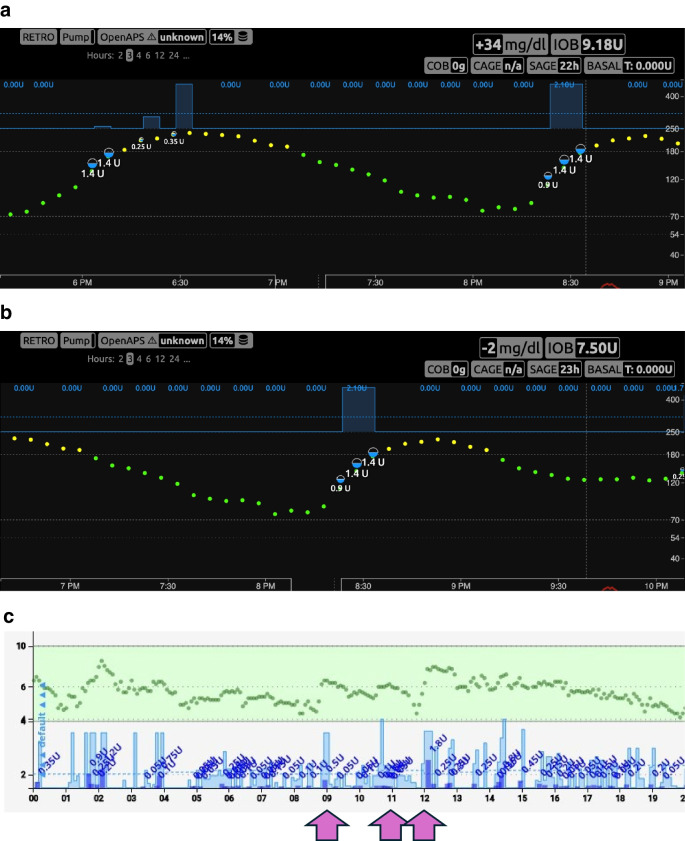
Example of insulin delivery without meal announcement in users with long-standing type 1 diabetes. (**a**, **b**) Nightscout data demonstrating insulin delivery using the SMB feature within AndroidAPS with insulin Lyumjev in response to unnanounced meals: a 30 g carbohydrate unannounced meal at 18:00 hours (**a**) and a 40 g carbohydrate unannounced meal at 20:10 hours (**b**). Time is denoted on the *x*-axis and glucose (in mg/dl) on the *y*-axis. Green dots indicate CGM readings within range, and yellow dots indicate readings above range, as set per the user. (**c**) Nightscout day report with data demonstrating insulin delivery using SMB within iAPS with insulin Lyumjev in response to carbohydrate intake at 09:00 hours (25 g), 11:00 hours (60 g) and 12:00 hours (40 g). Time is denoted on the *x*-axis and glucose (in mmol/l) on the *y*-axis. Green circles indicate CGM readings; dark blue bars indicate bolus insulin with corresponding amounts delivered by SMB. Light blue bars indicate basal insulin delivery. CAGE, cannula age (time elapsed since the last pump set insertion, used to monitor site degradation); COB, carbs on board (the estimated impact of recently consumed carbohydrates still affecting glucose levels); IOB, insulin on board (the amount of active insulin still acting in the body from previous dosing); SAGE, sensor age (time since the current CGM sensor was started, relevant for evaluating sensor accuracy and performance). This figure is available as part of a downloadable slideset

**Fig. 2:**
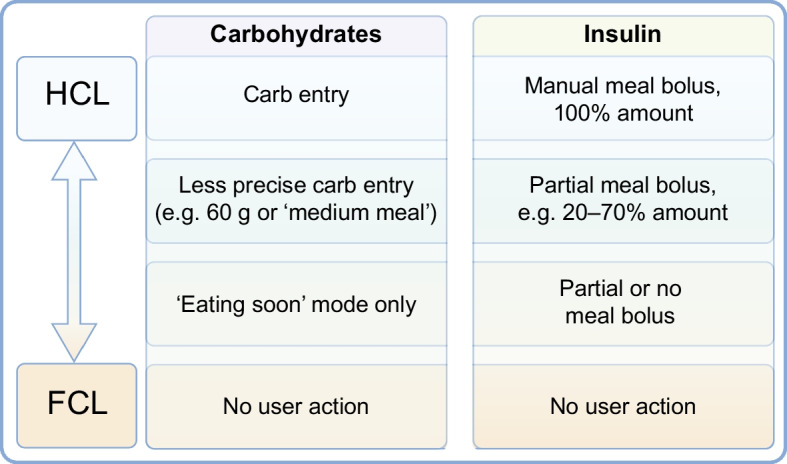
Stepwise pathway towards AID without meal announcement as conceptualised in the OS-AID framework. The hierarchy outlines the progression from current HCL systems towards more advanced automation without meal announcements. The framework supports bi-directional flexibility, allowing individuals to move both upwards and downwards within the hierarchy depending on changing needs, preferences or life circumstances. This allow users to choose their preferred level of operation in relation to meal announcements when desired. A menu of system configurations ensures a user-centred approach tailored to individual needs, preferences and clinical circumstances. The model supports a flexible, user-centric approach rather than a one-size-fits-all trajectory. This figure is available as part of a downloadable slideset

As these features were adopted, some OS-AID system users started optimising their settings to use their systems without any carbohydrate announcements or meal boluses, as shown in Fig. 1 and electronic supplementary material (ESM) Video 1 [28]. ESM Video 1 shows a time-lapse video (44 s) showing 3.5 h of Nightscout data, illustrating the operation of AndroidAPS in managing an unannounced meal. Others have taken a hybrid approach where they might announce a general carbohydrate estimate but do not enter a bolus or partial small bolus to initiate insulin activity at the front of the meal, leaving the system to address the rest of any insulin needed. As OS-AID systems do not adapt to meal announcements, users are not required to stick to the same patterns of user inputs and can thus flexibly choose on a meal-to-meal basis whether they employ an unannounced meal approach or anything along the spectrum of fewer user inputs or less-precise inputs than exact carbohydrate counts and manual meal boluses, as in most current traditional AID systems (Fig. 2). This flexibility represents a key distinction from commercial AID systems and has enabled real-world experimentation with a wide spectrum of user choices (Fig. 2).

Evidence shows that OS-AID systems are safe and effective, improving quality of life and lived experiences of people with diabetes [2]. Real-world studies consistently report improvements in glycaemic metrics, including increased TIR and reduced HbA_1c_ levels, without increased risk of hypoglycaemia [6, 29–33]. Further reports also highlight improved psychosocial outcomes using OS-AID systems [34–38]. A landmark RCT by Burnside et al [39], evaluating the OpenAPS algorithm on an Android device, provided compelling evidence supporting the safety and efficacy of these systems. The Tidepool Loop algorithm, also originating from the #WeAreNotWaiting community, has since received regulatory clearance from the US Food and Drugs Administration under the interoperability framework for AID systems, marking a significant milestone in the recognition of user-developed technologies and interoperable algorithms [40]. This provides encouraging support for the rigour and safety inherent in open-source approaches and sets a precedent for the design and approval of future AID systems.

Recent evidence has also demonstrated the potential of the AAPS using a simplified prandial bolus strategy that eliminates the need for carbohydrate counting [41]. In this approach, participants were trained to enter a standardised meal bolus equivalent to 50 g of carbohydrates during AID use. This was paired with a fixed insulin dose, individually tailored to account for the participant’s typical meal size recorded during a 7 day run-in period, and supplemented with correction insulin using a sliding scale. In a randomised cross-over study involving 32 adults, each undergoing 2 week intervention phases, this simplified HCL approach resulted in improved TIR glycaemic outcomes compared with conventional open-loop therapy (79.21% ± 4.84 vs 73.40% ± 9.98%).

The international consensus statement on OS-AID, published in *The Lancet’*s ‘100 Years of Insulin’ special issue, affirms the safety, effectiveness and psychosocial benefits of these technologies [2]. This consensus emphasises the importance of ethical considerations, advocating for informed, shared decision-making between clinicians and people with diabetes, and encourages transparent discussions to support user autonomy. It further calls for the development of collaborative frameworks among healthcare professionals (HCPs), regulators and the open-source community to safely and responsibly integrate these innovations into standard clinical care.

Importantly, the current accessibility of unannounced meal features unique to the open-source community parallels the situation where AID systems were initially inaccessible commercially or only available through clinical trials. While several commercial manufacturers are currently developing and testing AID systems without meal announcements, none have yet received regulatory clearance for this use. As such, OS-AID systems presently offer the only real-world access to unannounced meals, mirroring how open-source communities previously provided early access to AID systems before their commercial availability expanded.

## OS-AID without meal announcements: evidence and experience

Recent data from two clinical trials underscore the potential of OpenAPS systems to safely and effectively maintain glucose levels within a healthy range in the absence of meal announcements.

The Pancreas4ALL study was a randomised, open-label, cross-over residential camp study conducted in 16 adolescents with type 1 diabetes, comparing different versions of the AAPS system, including a no-meal announcement mode [4]. Participants (mean age 17 years, diabetes duration 5.9 years) cycled through three 3 day periods using a modified and locked version of AAPS in randomised order: quantitative carbohydrate entry; qualitative meal announcement; and no meal announcement. The primary outcomes were feasibility and safety, measured by system control time and hypoglycaemia incidence (glucose <3.0 mmol/l or 54 mg/dl). The system controlled glucose 95% of the time across all modes, with minimal hypoglycaemia (mean 0.72%), with quantitative carbohydrate entry showing a slightly higher rate (1.05%) than either qualitative meal announcement or no announcement (both 0.0%, *p*=0.05). This feasibility study, with a small sample size, was not powered to detect statistical differences and no notable differences were observed in TIR, mean glucose, insulin doses or daily carbohydrate intake. Mean TIR ranged from 79.9% to 83.3%, and no serious adverse events occurred.

Building on this, the CLOSE-IT trial (preliminary results presented at Advanced Technologies and Treatments or Diabetes 2025) evaluated AAPS over a longer duration in adults aged 18–70 years with type 1 diabetes [3, 42]. This randomised, open-label, parallel-group, non-inferiority study is the longest to date of an AID system without meal announcements in real-world conditions. After a 12 week run-in phase with meal announcements, participants were then randomised 1:1 to discontinue or continue meal announcements for 12 weeks. At study end, mean TIR was 66 ± 8% for unannounced meals vs 69 ± 13% for meal announcements, compared with 69 ± 11% and 70 ± 9%, respectively, at the end of the run-in. The adjusted between-group difference was −2.2 percentage points (95% CI −6.2, 1.7), meeting the criterion for non-inferiority (*p*=0.009). No significant difference in carbohydrate intake was found between the groups. Adherence to the no-meal-announcement protocol was confirmed. Participants on a low-carbohydrate diet were excluded, possibly limiting the generalisability of the findings.

Despite the encouraging efficacy and safety outcomes of these studies, several practical challenges were noted (summarised in ‘Challenges and considerations’).

The findings align with limited real-world data (ESM Table 1) showing reduced user input and only modest declines from prior HCL use, though small datasets and potential self-reporting bias remain limitations (ESM Table 1) [43, 44].

## Challenges and considerations

As demonstrated in the prior sections, the removal of meal announcements is beneficial but is far from complete automation. Due to the slow action of insulin, implementing unannounced meals requires fast reaction times in insulin delivery following an unexpected glucose excursion. The higher the carbohydrate load the greater the glycaemic excursion and the more insulin delivery needed. Additionally, insulin delivery must be reduced rapidly as glucose stops rising. The ability to rapidly adapt to meals in this fashion requires short-term, low-latency controllers with safety parameters for CGM noise and excursions that may not be associated with meals. Currently, only OpenAPS provides this functionality. In addition, system performance is entirely dependent upon continuous, accurate CGM data, stable pump communications and intact insulin infusion. Gaps in sensor usage mean the system will not have the data to act upon, which is particularly problematic if a sensor change occurs before or during a glucose excursion. Pump disconnections, removal and site failure mean the system is not able to effectively deliver insulin.

The OpenAPS algorithm has more configurable parameters than any other AID system. These settings allow for unprecedented levels of customisation that are ideally optimised by individuals with expertise in diabetes physiology, the control algorithm and human–machine interaction. The number of specialists with this level of expertise is limited but many healthcare providers can be trained. Many of the setting changes are straightforward, such as enabling SMBs, UAMs and allowing a larger microbolus during a significant glucose excursion. Where possible physiological settings such as basal, carbohydrate ratio and ISF should be true to the individual’s needs. Additional insights are available to providers through publications [2] and extensive documentation by the open-source community [26].

While the initial set-up may carry complexity, the use of a fully configured OS-AID system without meal announcement can be operationally simpler than most commercial systems. The user must be wearing a CGM device and pump, and be within Bluetooth low-energy (BLE) communication distance of their rig or smartphone. If these items remain connected, the only interaction required from the user is reporting a sudden change in insulin needs (e.g. activity or illness).

With this increased degree of automation, it can often be difficult to remember to announce sudden changes in insulin sensitivity, particularly when they occur less frequently than meals. Temporary target adjustments are commonly used for this purpose, although other settings can also be adjusted for short-term periods. Additionally, behavioural factors influence overall outcomes: the system will generally perform better when meal frequency, size and carbohydrate content are reduced. While this may promote healthier eating patterns, it can also inadvertently reduce engagement in physical activity, as exercise often requires additional interaction with the system to activate temporary targets or overrides.

Even with these design features, sudden unexpected changes in insulin sensitivity that occur after prandial insulin delivery may have a significant impact on postprandial glucose levels. Exercise is one such event that is challenging to predict beforehand.

A limitation of current insulin-only systems is the delay and duration of subcutaneous insulin action, which makes unannounced high-carbohydrate meals challenging for any control algorithm. Adjunctive therapies, such as sodium–glucose cotransporter 2 (SGLT2) inhibitors, glucagon-like peptide 1 (GLP-1) receptor agonists, amylin analogues such as pramlintide, and glucagon, may further enhance the effectiveness of current systems [45–47]. These agents can suppress postprandial glucagon secretion, slow gastric emptying and therefore mitigate meal-related glucose excursions. The co-administration of amylin analogues with meal announcements has given results comparable with those of AID plus carbohydrate counting [48, 49]. Finally, dual-hormone systems aim to safely allow more aggressive insulin dosing by adding its counterpart glucagon to prevent hypoglycaemia [48, 49].

## Clinician perspective

The use of AID systems in HCL mode has improved outcomes. However, as highlighted above, bolus burden and user behaviour to achieve optimal outcomes remains a limitation. OpenAPS without meal announcements can overcome some of these challenges to improve glycaemic outcomes with reduced burden. However, these systems require some work to initiate and personalise before they can function optimally without meal announcement. A challenge is ensuring that HCPs are adequately educated to effectively train, utilise and adjust these systems, especially as HCP training for AID systems has been driven by industry-supported programmes in most regions, meaning that open-source system use has not been addressed in sufficient detail. There is no dedicated support line for OS-AID systems; instead, troubleshooting support is primarily community-driven through established responsive online channels, including national and international peer-support forums. While strong community support is available, not all people with type 1 diabetes or HCPs may be open to using or signposting this form of support. HCPs can draw on resources, referenced previously, to guide their practical and ethical involvement in supporting OS-AID users [2, 26, 50–52].

The complexity inherent in their initial settings and tuning further limits the effective use of OS-AID systems. Nevertheless, OS-AID without the need for meal announcements exemplify promising innovation, demonstrating evidence for efficacy and safety. As clinicians, our current role is primarily supportive: encouraging and assisting individuals who choose to use these advanced technologies. While OS-AID systems are technically available to anyone, concerns are often raised that these systems are only feasible for highly motivated or technically skilled users. However, data from the international OPEN project suggest a more diverse user base [35, 38, 53–55]. Many individuals without formal technical backgrounds have successfully built and used OS-AID systems, supported by comprehensive community documentation and peer-support. Rather than technical aptitude, the more relevant preconditions for use include the ability to understand that these systems have usually not received regulatory clearance, the willingness to make an informed decision, and the capacity to invest time during set-up and occasional system maintenance. Clinical contraindications may mirror those for commercial AID systems, with additional consideration of individual autonomy, digital literacy and support networks [2]. Personalisation of OS-AID systems is key to their optimal function; however, if HCPs do not know how to guide this process, it can be a significant barrier. This limits the potential benefits of use and highlights the need for simplicity in set-up and adjustment, as well as broader training and accessible resources for clinicians.

Transparency is pivotal for all AID algorithms, and this is a unique strength of open-source systems, where the code is fully accessible for anyone to review. As with all AID systems, there have been reports of acute complications and adverse events in both clinical trials and post-market surveillance systems such as MAUDE and EUDAMED. OS-AID systems use the same commercial hardware for CGMs and pumps as commercial AID systems and are subject to the same hardware-related risks, with the addition of publicly documented issue tracking and fixes available in each system’s GitHub repository.

Clear, transparent communication from HCPs is essential to help individuals evaluate whether OS-AID is appropriate for their needs and circumstances. Concurrently, we have a valuable opportunity to learn from these early adopters. Such insights will inform future development and help shape more accessible solutions. Recently, one iOS variant of OpenAPS, Trio, has released a version focused on usability with a beautiful interface and simplified onboarding, improving accessibility for people with type 1 diabetes and providers alike. Such initiatives and learning from the open-source community could ultimately pave the way to enabling wider adoption as the simplicity in their technical set-up evolves.

While data are encouraging and highlight the potential for systems that do not require meal announcements to improve outcomes for a significant subset of people who are not able to bolus or enter meals regularly, the outcomes still remain short of time-in-tight range or time-in-normoglycaemia ranges being considered for AID and novel therapies [56]. We acknowledge that these observations are also based on early data and further robust clinical trials are needed to define the role of OS-AID without meal announcements in routine practice. Nevertheless, these initial experiences provide a strong foundation for further development and innovation highlighted below.

## Future directions and opportunities

Next-generation AID systems are expanding their automation beyond meals. Systems are being developed to anticipate or respond to exercise, stress, and hormone- and sleep-related variations [57–63]. Current AID systems offer user-initiated ‘exercise modes’ (e.g. by setting temporary glucose targets), with some open-source systems enabling subsequent adjustments to settings related to exercise [64]. Future designs aim for passive detection of physical activity via wearables or sensor patterns. Early studies integrate wearables to adapt insulin around exercise to prevent activity-related hypoglycaemia [65]. Incorporating circadian patterns and stress responses is a further nascent area, where future systems may adjust insulin dosing according to sleep stages or stress-hormone surges. There is also work to determine whether identifying patterns of hypoglycaemia may be useful for further adjusting system settings until glycaemic variability normalises. Given the concerns around higher risks of diabetic ketoacidosis (DKA) with AID systems, compared with SAPT, an additional avenue for future development is the integration of continuous ketone monitoring (CKM) into AID systems [66]. While CKM is not yet validated for routine DKA prevention and robust clinical thresholds are needed, its combination with glucose data could, in the future, help identify and prevent metabolic decompensation earlier. With ‘smarter’ algorithms, faster-acting insulins and adjunctive medications, the gap to full automation steadily becomes narrower. Any new technology should allow the majority of users to achieve consensus glucose targets without user input. The use of artificial intelligence such as large language models and other advanced computational tools may also support the development and refinement of AID systems.

## Conclusion

Early adopters in the #WeAreNotWaiting community are reaching glycaemic goals using OS-AID without meal announcements in early reports. While these systems do not yet support such use for everyone, they represent a significant step towards greater automation. This is not yet common with commercial AID systems but is on the horizon with future iterations of commercial technology. Current evidence for OS-AID without meal announcements is drawn from a limited number of well-designed but early studies, complemented by encouraging findings from selected user experiences. Further robust studies are needed to determine generalisability with detailed practical considerations for HCPs, adoption and success. Nevertheless, the first real-world implementations of AID use without meal announcements have emerged from the diabetes community without invoking artificial intelligence or machine learning. These systems rely on user insight and personal configuration, underscoring the importance of involving experienced users in the development of future technologies. As the field moves towards more advanced automation, it is essential for clinicians, researchers and industry stakeholders to remain engaged with these grassroots innovations, and for commercial organisations to pull inspiration and motivation to bring these innovations to market more quickly. Doing so will help ensure the safe, equitable and person-centred implementation of the next generation of care for all people living with diabetes.

## Supplementary Information

Below is the link to the electronic supplementary material.

ESM (PDF 104 KB)

ESM Video 1 (MP4 4309 KB)

Slideset of figures (PPTX 365 KB)

## Abbreviations

AID: Automated insulin delivery
CGM: Continuous glucose monitoring
CKM: Continuous ketone monitoring
CSII: Continuous subcutaneous insulin infusion
DKA: Diabetic ketoacidosis
FCL: Fully closed-loop
GLP-1: Glucagon-like peptide 1
HCL: Hybrid closed-loop
HCP: Healthcare professional
ISF: Insulin sensitivity factor
OS-AID: Open-source AID
PLGS: Predictive low-glucose suspend
SAPT: Sensor-augmented pump therapy
SMB: Supermicrobolus
TIR: Time in range
UAM: Unannounced meal
