# Supplementary material for: Fully closed-loop systems: can people with type 1 diabetes just do it? Insights from open-source systems
Source: Diabetologia. 2026 Jan 19;69(3):557–67. doi: 10.1007/s00125-025-06644-8 (PMC12881006; doi:10.1007/s00125-025-06644-8)
Supplement: Supplementary file 1 — ESM (PDF 104 KB) [file 125_2025_6644_MOESM1_ESM.pdf]

ESM Video 1.

Time-lapse video (44 seconds) showing 3.5 hours of Nightscout data, illustrating the operation of AndroidAPS in managing an unannounced meal. The example depicts insulin (Lyumjev) delivery in response to ingestion of 75 g carbohydrate without meal announcement or bolus administration in an individual with long-standing type 1 diabetes who was also receiving adjunctive therapy with Tirzepatide and using ultra-rapid insulin (Lyumjev). The narration describes the use of Android APS with Boost feature, which delivers an early, larger super microbolus in response to the unannounced meal via a multi-phase algorithm. (This example is provided for illustrative purposes only).

ESM Table 1: This table summarises the key glycaemic metrics from survey, cross-over study and recent trial data with OpenAPS-derived OS-AID systems.[45]

	**Survey**			**Pancreas 4ALL**			**Close-IT Trial**	
	Online survey of open-source users with T1D			Randomised cross-over camp study using AndroidAPS in children with T1D			Randomised control trial, multi-site study using AAPS in adults with T1D	
**Use mode**	HCL	Occasional bolus	No meal bolus	HCL	MA	No MA	HCL	No MA
**n=**	51	27	15	16	16	16	37	36
**TAR (%)**	10.5	13.4	10.7	8.1	14.4	12.5	32	29
**TIR (%)**	86.2	83.3	86.9	83.3	79.9	81	69	66
**TBR (%)**	3.3	3.3	2.4	4.6	2.4	1.4	1.6	1.7
