# Supplementary material for: Fully closed-loop systems: can people with type 1 diabetes just do it? Insights from open-source systems
Source: Diabetologia. 2026 Jan 19;69(3):557–67. doi: 10.1007/s00125-025-06644-8 (PMC12881006; doi:10.1007/s00125-025-06644-8)
Supplement: Supplementary file 3 — Slideset of figures (PPTX 365 KB) [file 125_2025_6644_MOESM3_ESM.pptx]

## Slide 1
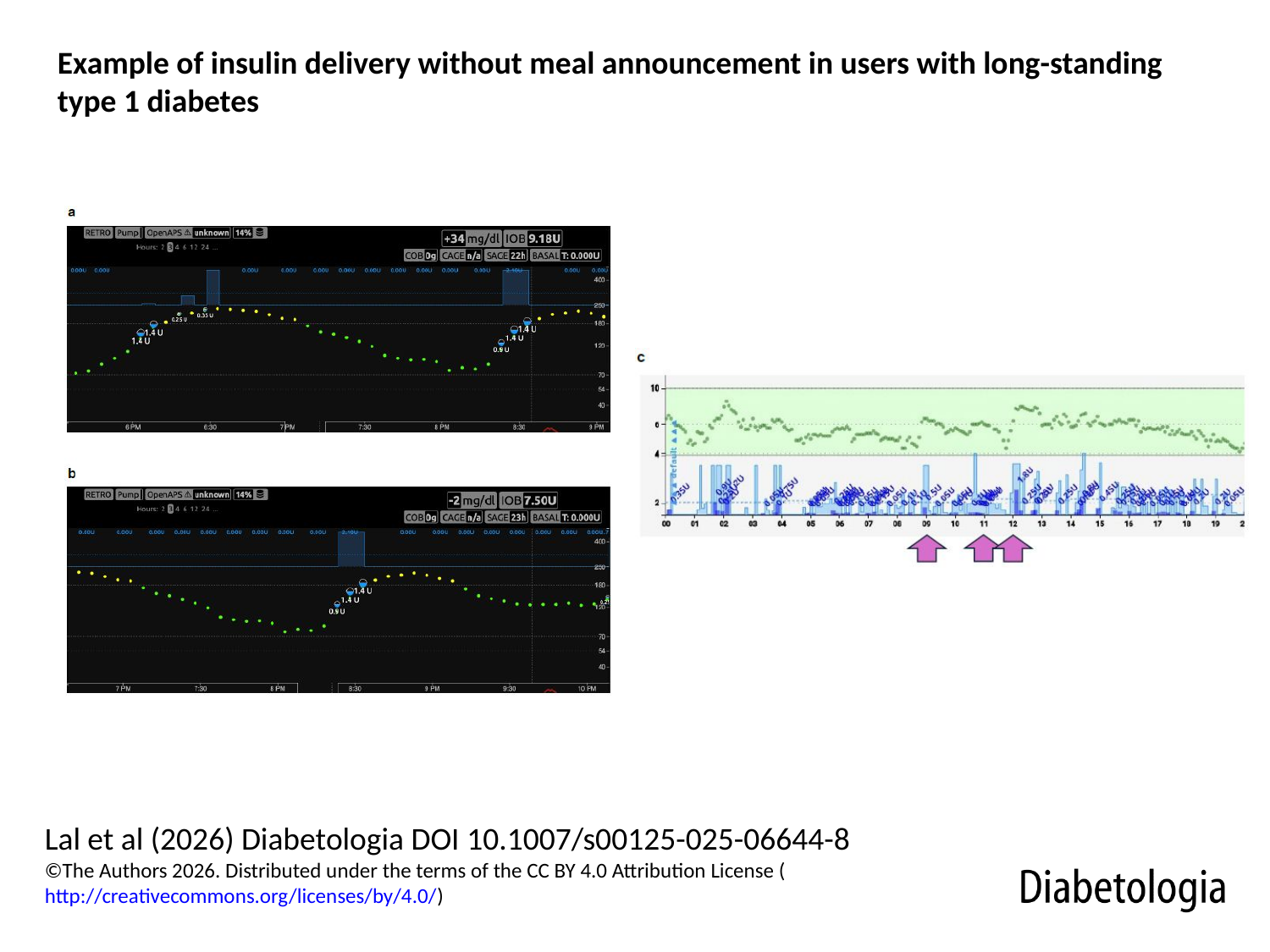

Example of insulin delivery without meal announcement in users with long-standing
type 1 diabetes
Lal et al (2026) Diabetologia DOI 10.1007/s00125-025-06644-8
©The Authors 2026. Distributed under the terms of the CC BY 4.0 Attribution License (http://creativecommons.org/licenses/by/4.0/)

## Slide 2
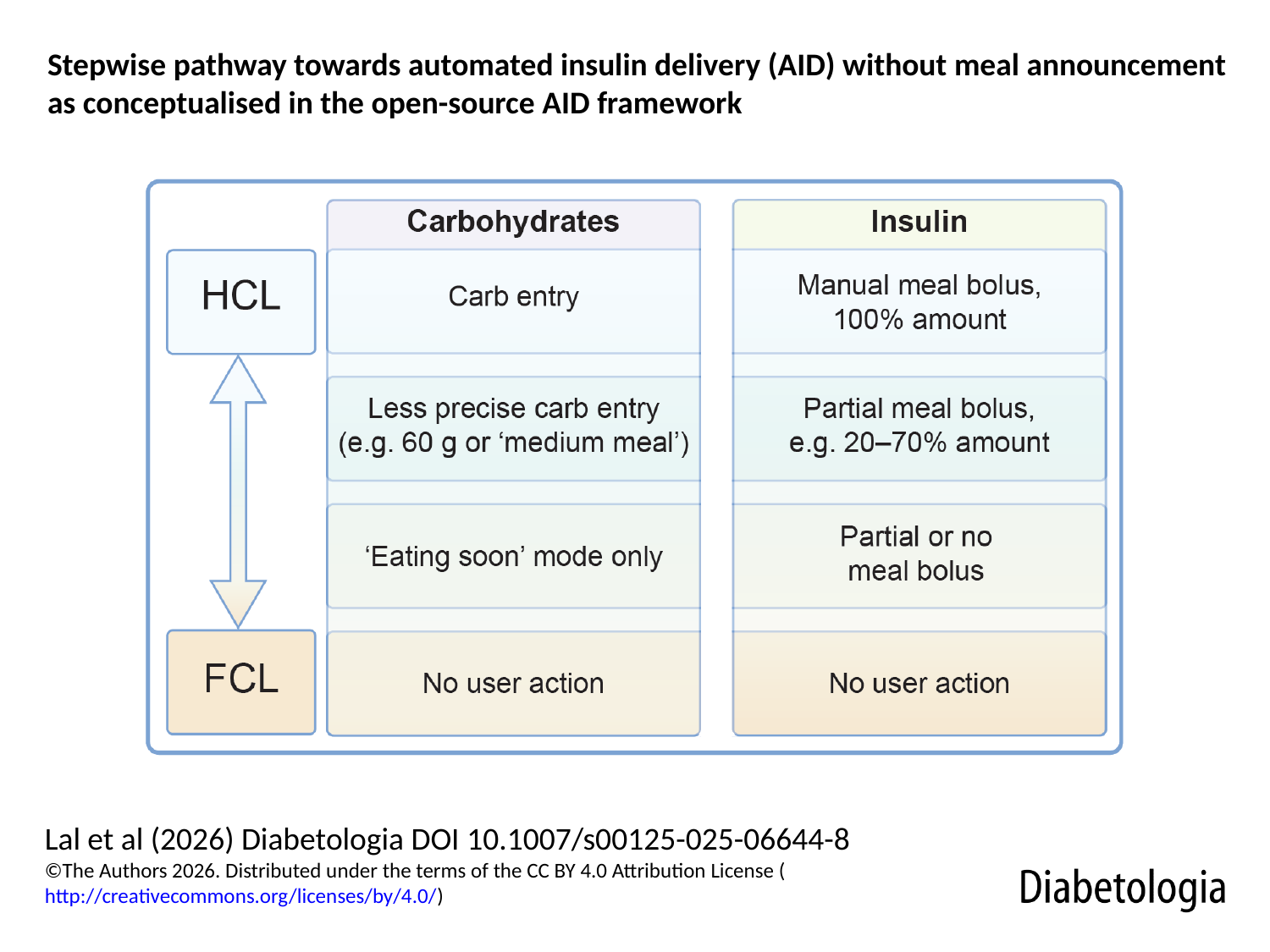

Stepwise pathway towards automated insulin delivery (AID) without meal announcement as conceptualised in the open-source AID framework
Lal et al (2026) Diabetologia DOI 10.1007/s00125-025-06644-8
©The Authors 2026. Distributed under the terms of the CC BY 4.0 Attribution License (http://creativecommons.org/licenses/by/4.0/)
